# Expression of endothelia and lymphocyte adhesion molecules in bronchus-associated lymphoid tissue (BALT) in adult human lung

**DOI:** 10.1186/1465-9921-10-97

**Published:** 2009-10-22

**Authors:** Nakaaki Kawamata, Baohui Xu, Hiroo Nishijima, Kohji Aoyama, Mayumi Kusumoto, Toru Takeuchi, Chuwa Tei, Sara A Michie, Takami Matsuyama

**Affiliations:** 1 Departments of Immunology, Graduate School of Medical and Dental Sciences, Kagoshima University, 8-35-1 Sakuragaoka, Kagoshima 890-8520, Japan; 2 Cardiovascular, Respiratory and Metabolic Medicine, Graduate School of Medical and Dental Sciences, Kagoshima University, 8-35-1 Sakuragaoka, Kagoshima 890-8520, Japan; 3 Environmental Medicine, Graduate School of Medical and Dental Sciences, Kagoshima University, 8-35-1 Sakuragaoka, Kagoshima 890-8520, Japan; 4 Department of Pathology, School of Medicine, Stanford University, Stanford, CA 94305, USA; 5 Department of Surgery, Kagoshima Kouseiren Hospital, Kagoshima, Japan

## Abstract

**Background:**

Bronchus-associated lymphoid tissue (BALT) is the secondary lymphoid tissue in bronchial mucosa and is involved in the development of bronchopulmonary immune responses. Although migration of lymphocytes from blood vessels into secondary lymphoid tissues is critical for the development of appropriate adaptive immunity, the endothelia and lymphocyte adhesion molecules that recruit specific subsets of lymphocytes into human BALT are not known. The aim of this study was to determine which adhesion molecules are expressed on lymphocytes and high endothelial venules (HEVs) in human BALT.

**Methods:**

We immunostained frozen sections of BALT from lobectomy specimens from 17 patients with lung carcinoma with a panel of monoclonal antibodies to endothelia and lymphocyte adhesion molecules.

**Results:**

Sections of BALT showed B cell follicles surrounded by T cells. Most BALT CD4^+ ^T cells had a CD45RO^+ ^memory phenotype. Almost all BALT B cells expressed α_4 _integrin and L-selectin. In contrast, 43% of BALT T cells expressed α_4 _integrin and 20% of BALT T cells expressed L-selectin. Almost all BALT lymphocytes expressed LFA-1. HEVs, which support the migration of lymphocytes from the bloodstream into secondary lymphoid tissues, were prominent in BALT. All HEVs expressed peripheral node addressin, most HEVs expressed vascular cell adhesion molecule-1, and no HEVs expressed mucosal addressin cell adhesion molecule-1.

**Conclusion:**

Human BALT expresses endothelia and lymphocyte adhesion molecules that may be important in recruiting naive and memory/effector lymphocytes to BALT during protective and pathologic bronchopulmonary immune responses.

## Background

The lower respiratory tract is continually exposed to a wide variety of airborne antigens and microorganisms. The generation of rapid, specific adaptive immune responses to inhaled antigens and pathogens is critical for survival. Unfortunately, in some inflammatory and infectious diseases, such as asthma and tuberculosis, these immune responses can damage the lungs and bronchi. Thus, the bronchopulmonary adaptive immune system is involved in the maintenance of health and the development of diseases of the lower respiratory tract.

In the initiation stage of an adaptive immune response, naive T cells migrate through blood vessel high endothelia venules (HEVs) into secondary lymphoid tissues, where they are stimulated by antigen-bearing dendritic cells. This leads to the generation of antigen-specific effector and memory T cells and B cells, which are released from the secondary lymphoid tissue into the bloodstream. In the effector stage of the adaptive immune response, some of the memory T cells and B cells migrate from blood vessels into nonlymphoid tissue(s) that contain the cognate antigens or pathogens [1-4]. The lower respiratory tract has two distinct types of secondary lymphoid tissues: bronchus-associated lymphoid tissue (BALT) and lymph nodes (LNs) [5-7]. The presence of BALT in adult mammals depends on species, antigen stimulation and age [6-8]. BALT is found in normal lungs of most healthy adult rabbits, rats, guinea pigs and old adult mice [5,6,9-13]. In contrast, the presence and frequency of BALT in normal lungs of healthy adult humans is controversial [5,14-20].

BALT is the site of initial presentation of inhaled antigens, which are transported from the bronchial lumen by specialized cells (likely M cells and/or dendritic cells) in the epithelia over the BALT, to naive T cells, which enter the BALT through blood vessel HEVs [21-26]. Intravascular lymphocytes must bind to the endothelial surface of HEVs in BALT and other secondary lymphoid tissues, prior to migrating through the vessel wall into the tissue [1,9,27]. Thus, the adhesion molecules expressed on the surface of the HEVs help determine which subsets of lymphocytes can migrate into the tissue. There are three distinct, tissue-selective combinations of adhesion molecules on HEVs in secondary lymphoid tissues. Most HEVs in peripheral LNs of mice and humans express peripheral node addressin (PNAd), but not mucosal addressin cell adhesion molecule-1 (MAdCAM-1) and vascular cell adhesion molecule-1 (VCAM-1). Most HEVs in small intestine Peyer's patches (PP) express MAdCAM-1, but not VCAM-1 and luminal PNAd. Most HEVs in mouse BALT express PNAd and VCAM-1, but not MAdCAM-1 [1,9,27]. *In vivo *studies from our laboratory indicate that migration of B cells and naive T cells into mouse BALT largely depends on endothelial PNAd and its lymphocyte ligand L-selectin, while migration of memory T cells into BALT requires endothelial VCAM-1 and lymphocyte α_4 _integrin [9]. However, the adhesion molecules that are highly expressed on HEVs in human BALT are not known.

In this study, we evaluated BALT tissue from lobectomy specimens of 17 human adults with lung carcinoma. We found that BALT HEVs expressed PNAd and VCAM-1 but not MAdCAM-1. Most BALT B cells and some T cells expressed L-selectin and α_4 _integrin. Most BALT CD4^+ ^T cells had a memory phenotype. These results suggest that human BALT expresses endothelia and lymphocyte adhesion molecules that may be important in recruiting naive and memory/effector lymphocytes to BALT, where they may be involved in the initiation of bronchopulmonary immune responses to inhaled antigens and pathogens.

## Methods

### Tissue collection, frozen sectioning and histologic identification of BALT

Bronchus tissue was obtained from 49 patients who underwent lobectomy for lung carcinoma at Kagoshima Kouseiren Hospital, Kagoshima, Japan. Patients with pulmonary inflammatory disorders, such as asthma, chronic obstructive pulmonary disease, rheumatoid lung, and post-obstructive pneumonia due to tumor, were excluded from the study. Tissue samples, each ≥2 cm in maximum dimension, of bronchus and surrounding lung were taken from at least 2 nonadjacent sites in each lobectomy specimen. The sampling was carefully performed to exclude tumor. Each tissue sample was infused with a 1:1 mixture of OCT compound (Sakura Finetek, Tokyo, Japan) and phosphate-buffered saline (PBS), and frozen in OCT compound. A frozen section was cut, fixed in methanol, stained with hematoxylin and examined by light microscopy for "classic" BALT, as defined using the histologic criteria originally described and illustrated by Bienenstock and colleagues [5]. These criteria are listed in the first paragraph of the Results section. If "classic" BALT was identified on the hematoxylin-stained section, additional sections were cut, fixed in acetone, and stored at -20°C for immunostaining. If there was no BALT on the hematoxylin-stained section, we continued to cut and examine sections at 100 μm intervals until BALT was found, or the block was cut through.

Small intestine PPs, obtained from ileocecal resection specimens from 3 patients with colon carcinoma, were frozen in OCT compound. Frozen sections were cut, fixed in acetone, and stored at -20°C for immunostaining.

### Antibodies and other reagents

Mouse and rat monoclonal antibodies (mAbs) used in this study are listed in Table 1. Biotin-anti-mouse IgG polyclonal antibody was purchased from eBioscience (San Diego, CA). Biotin-anti-rat IgM polyclonal antibody and peroxidase-streptavidin were obtained from Jackson ImmunoResearch Laboratories (West Grove, PA). AEC substrate kit and Alexa Fluor 546-streptavidin were from Vector Laboratories (Burlingame, CA) and Molecular Probes (Eugene, OR), respectively.

**Table 1:** Monoclonal Antibodies Used for Tissue Immunostaining

mAb	Clone	Species Isotype	Source
CD3	UCHT1	mouse IgG1	BD Biosciences
CD4	RPA-T4	mouse IgG1	eBioscience
CD8	RPA-T8	mouse IgG1	eBioscience
CD19	HIB19	mouse IgG1	BD Biosciences
CD45RA	HI100	mouse IgG2b	BD Biosciences
CD45RO	UCHL1	mouse IgG2a	eBioscience

CD11a/LFA-1	HI111	mouse IgG1	eBioscience
CD49d/α_4 _integrin	9F10	mouse IgG1	eBioscience
CD62L/L-selectin	Dreg 56	mouse IgG1	BD Biosciences

CD31/PECAM-1	WM59	mouse IgG1	eBioscience
CD54/ICAM-1	HA58	mouse IgG1	eBioscience
CD102/ICAM-2	CBR/IC2/2	mouse IgG2a	Caltag Laboratories
MAdCAM-1	MMC3	mouse IgG1	Dina Washington, Genentech
PNAd	MECA79	rat IgM	Dr. Eugene C. Butcher, Stanford University
CD106/VCAM-1	D8	mouse IgG1	Dr. Takami Matsuyama, Kagoshima University

Species and isotype matched negative controls	N/A	mouse IgG1	eBioscience
	N/A	mouse IgG2a	eBioscience
	N/A	mouse IgG2b	eBioscience
	OZ42	rat IgM	Dr. Leslie Pickford

### Immunohistochemical staining

Acetone-fixed frozen sections of BALT and PP were incubated with mAb against CD31, MAdCAM-1, VCAM-1 or PNAd, or with a species- and isotype-matched negative control mAb, at room temperature for 1 hour (hr). Sections were rinsed in PBS, incubated with biotin-anti-mouse IgG (to detect CD31, MAdCAM-1 or VCAM-1) or biotin-anti-rat IgM (to detect PNAd) at room temperature for 30 minutes (min), rinsed in PBS, and incubated with peroxidase-streptavidin for 30 min. After a final PBS wash, slides were incubated with Vector AEC substrate (per manufacturer's instructions), stained with hematoxylin, and cover-slipped. Sections were evaluated by light microscopy.

### Immunofluorescence (IF) staining

To evaluate expression of ICAM-1 and ICAM-2 on blood vessel endothelia, frozen sections of BALT and PP were sequentially incubated with anti-ICAM-1 mAb, anti-ICAM-2 mAb or negative control mAb (1 hr), biotin-anti-mouse IgG (30 min), and Alexa Fluor 546-streptavidin (30 min).

To identify B cells and T cells, frozen sections of BALT and PP were sequentially incubated with anti-CD19 mAb (1 hr), biotin-anti-mouse IgG (30 min), 10% normal mouse serum (30 min), and Alexa Fluor 546-streptavidin combined with FITC-anti-CD3 mAb (30 min). CD4^+ ^cells and CD8^+ ^cells were identified by incubating the sections with PE-anti-CD4 and FITC-anti-CD8 mAbs (30 min). Naive CD4^+ ^cells were identified by staining with PE-anti-CD4 and FITC-anti-CD45RA mAbs, while memory CD4^+ ^cells were identified by staining with PE-anti-CD4 and FITC-anti-CD45RO mAbs. We counted CD4^+^CD45RA^+ ^cells (naive CD4^+ ^T cells) and CD4^+^CD45RO^+ ^cells (memory CD4^+ ^T cells) in BALT from 7 randomly selected patients, and calculated the percent of CD4^+ ^T cells with naive or memory phenotype.

To evaluate co-expression of VCAM-1 and PNAd on BALT HEVs, sections were sequentially incubated with anti-VCAM-1 mAb (1 hr), biotin-anti-mouse IgG in 4% normal human serum (30 min), 10% normal rat serum (30 min), and FITC-anti-PNAd mAb combined with Alexa Fluor 546-streptavidin (30 min). In 5 randomly selected patients, we determined the percentage of PNAd^+ ^HEVs that expressed VCAM-1.

To evaluate expression of adhesion molecules on BALT lymphocytes, sections were incubated with FITC-anti-CD3 mAb combined with PE-anti-LFA-1, PE-anti-α_4 _integrin, or PE-anti-L-selectin mAb (30 min). We counted total T cells (CD3^+^) and T cells expressing LFA-1, α_4 _integrin or L-selectin in BALT from 6 randomly selected patients, and calculated the percentage of T cells that expressed each adhesion molecule. In a similar manner, we determined the proportion of B cells (CD3^- ^lymphocytes in the B cell follicle) that expressed each adhesion molecule.

For each tissue, primary mAbs were replaced by conjugation-, species- and isotype-matched negative control mAbs. All slides were viewed and imaged using a confocal microscope.

### Ethics

The ethics committee of the Graduate School of Medical and Dental Sciences, Kagoshima University, Japan approved this study. Each patient gave written informed consent to use tissue for research.

### Statistical analysis

All data are expressed as mean ± standard derivation (SD). Chi-square test was used to evaluate the difference in the frequency of BALT between groups. *P *< 0.05 was considered to be statistically significant.

## Results

### Detection of BALT in adult human lung

**Figure 1:**
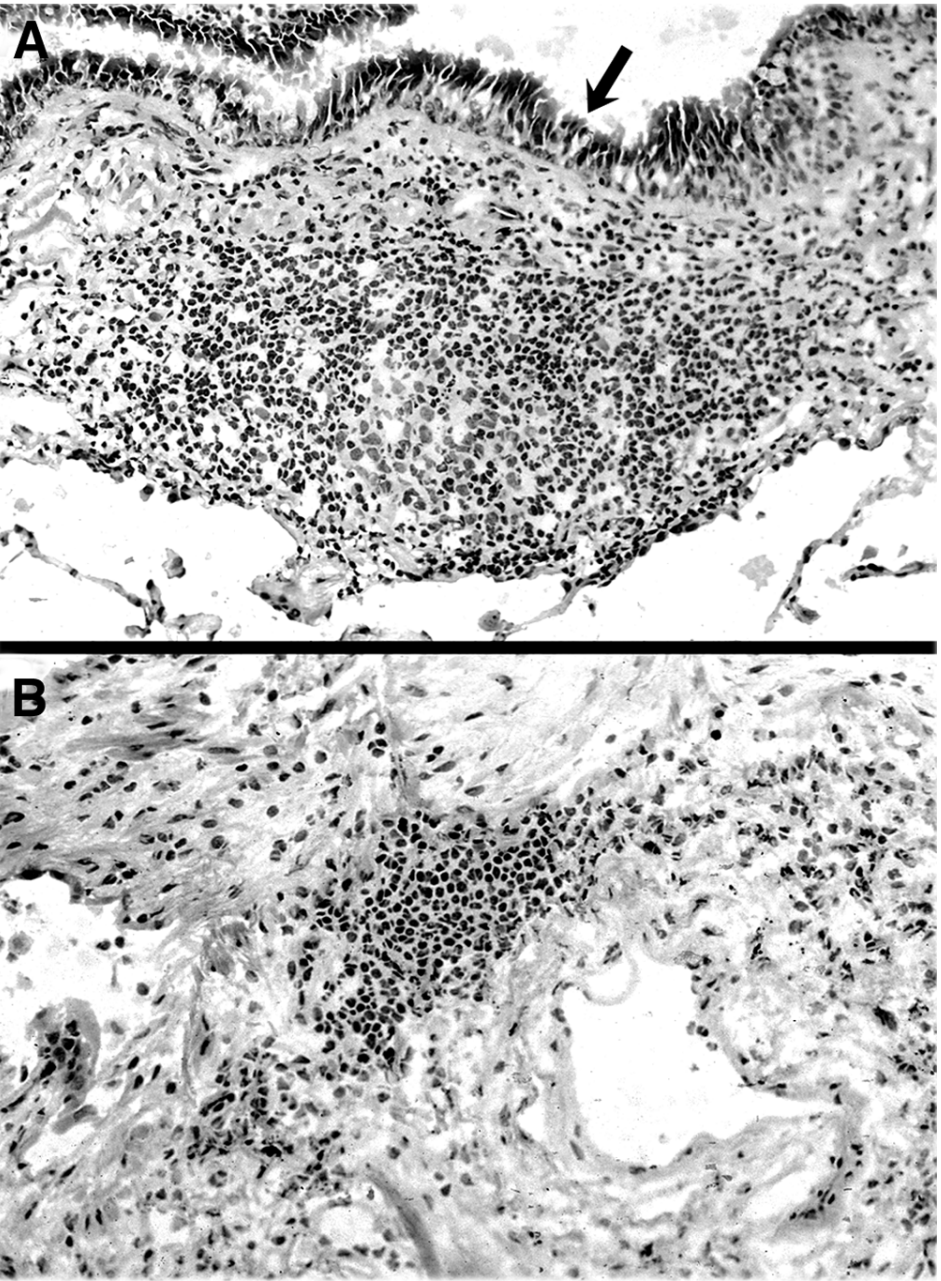
**Lymphoid tissue in human lung**. **(A)**: Bronchus-associated lymphoid tissue (BALT). There is a well demarcated submucosal lymphoid follicle in the wall of a bronchus. The arrow is in the bronchial lumen, pointing to the bronchial epithelia. **(B)**: Nonspecific lymphoid aggregate. There is a small, poorly demarcated lymphoid aggregate in a small focus (<1 mm diameter) of scar tissue in the lung interstitium. The aggregate is not associated with a bronchus. This aggregate does not meet the histologic criteria for BALT and is not included in this study. (hematoxylin and eosin stained frozen sections; original magnification 200×).

We examined hematoxylin-stained frozen sections of bronchi from lung carcinoma lobectomy specimens for BALT. We used histologic criteria that were originally proposed by Bienenstock and are extensively described in the human histopathology literature [5,28,29]. Specifically, BALT: 1) consists of lymphoid follicle(s) in the bronchial mucosa; 2) is fairly well circumscribed, without inflammation or damage of the adjacent bronchus tissue, 3) has prominent HEVs, and no or small germinal centers; 4) may contain pigment-filled macrophages, but no neutrophils or eosinophils; and 5) lacks evidence of significant epithelial injury. If BALT was identified on the hematoxylin-stained section (Fig. 1A), we immediately cut additional sections from the frozen tissue block for immunohistology staining. In some specimens, we found a few small, poorly demarcated lymphoid aggregates in the pulmonary parenchyma (Fig. 1B). These aggregates, which were not associated with a bronchus, were not analyzed in this study.

We identified BALT on hematoxylin-stained frozen sections of bronchus from 17/49 (35%) lung lobectomy specimens (Fig. 1A). BALT was identified in 40% of the lobectomy specimens from men and 26% of the specimens from women. Current smokers (42%) were more likely to have BALT than were previous smokers (25%) or non-smokers (29%). BALT was slightly more common in patients over the age of 70 years (37%) than in those 70 years or younger (32%). BALT was found in 43% of patients with squamous cell carcinoma and 31% of patients with adenocarcinomas. None of these differences were statistically significant (*p *> 0.05 for all factors, Chi-square test).

### Adhesion molecules on BALT HEVs

To determine which adhesion molecules are expressed on BALT HEVs, we stained frozen sections of BALT with mAbs against endothelia adhesion molecules that are expressed by HEVs in secondary lymphoid tissues. These adhesion molecules include PNAd, which is highly expressed by HEVs in peripheral LNs and mouse BALT; MAdCAM-1, which is expressed by HEVs in small intestine PPs; VCAM-1, which is highly expressed by HEV endothelia in mouse BALT; and ICAM-1 and ICAM-2, which are expressed by HEVs in LNs, PPs and mouse BALT [1,9]. For comparison to BALT, we also stained sections of PP.

**Figure 2:**
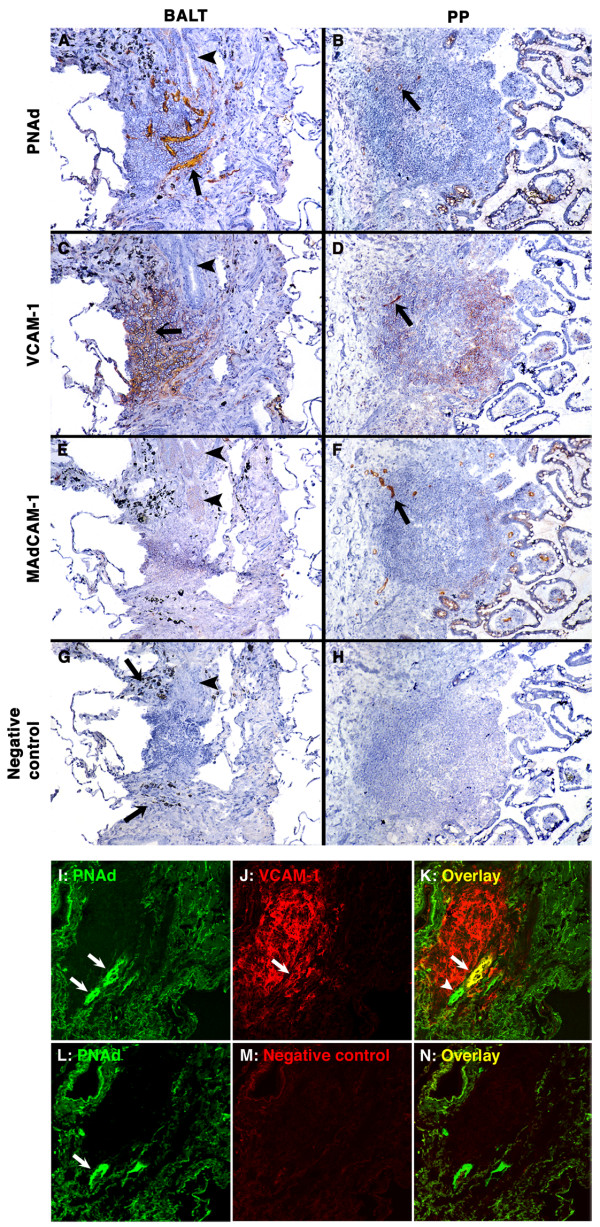
**PNAd and VCAM-1 are expressed on HEVs in human BALT**. **(A-H)**: Semi-serial frozen sections of BALT (A, C, E, G; arrowheads highlight the bronchial epithelium) and PP (B, D, F, H) were stained with mAbs against PNAd (A, B), VCAM-1 (C, D) and MAdCAM-1 (E, F), or with an isotype-matched negative control mAb (G, H). (A and B) PNAd is expressed on BALT HEVs (A; arrow highlights one HEV) and on a few vessels in PP (B; arrow). (C and D) VCAM-1 is expressed on BALT HEVs (C; arrow highlights one HEV) and on one vessel in PP (D; arrow). VCAM-1 is also expressed on non-endothelial cells in BALT and PP. (E and F) MAdCAM-1 is expressed on vessels in PP (F; arrow), but not in BALT (E). (G and H) Mouse IgG1 (for anti-VCAM-1 and MAdCAM-1) and rat IgM (for anti-PNAd mAb; not shown) negative control mAbs did not stain HEVs in BALT (G) and PP (H). (A, C, E, G) The black granular material (highlighted by arrows in G) is carbon pigment in macrophages. (immunoperoxidase stains. original magnification 100×). **(I-N)**: Semi-serial frozen sections of BALT were stained with mAbs against PNAd (green) and VCAM-1 (red) (I-K) or with anti-PNAd mAb (green) and negative control mAb (L-N) by two-color immunofluorescence. Anti-PNAd mAb gave strong staining of BALT HEVs (I, L; arrows). The anti-VCAM-1 mAb stained HEVs (J; arrow) and non-endothelial cells in BALT. PNAd^+^VCAM-1^+ ^(K; arrow) and PNAd^+^VCAM-1^- ^(K; arrowhead) HEVs were found in BALT. The negative control mAb for VCAM-1 did not stain BALT (M, N). (two-color immunofluorescence stains. original magnification 200×).

There was strong expression of PNAd on all HEVs in BALT (Fig. 2A) and on a few small vessels in PP (Fig. 2B). VCAM-1 was expressed on many HEVs in BALT, on rare HEVs in PP, and on many non-endothelial cells in BALT and PP (Figs. 2C, D). There was no staining for MAdCAM-1 on BALT HEVs in any of the patients (Fig. 2E). Bronchial venules that were not in BALT did not express PNAd, VCAM-1 or MAdCAM-1 (not shown), indicating that the bronchi were not chronically inflamed. As expected, there was strong staining for MAdCAM-1 on PP HEVs (Fig. 2F) [30]. ICAM-1 and ICAM-2 were strongly expressed on HEVs in BALT and PP (data not shown). Negative control mAbs did not stain vascular endothelia in BALT or PP sections (Figs. 2G, H).

VCAM-1 is known to be expressed by a variety of non-endothelial cells, including follicular dendritic cells and blood vessel pericytes, in human secondary lymphoid tissues [31]. Because of this extensive expression, it is difficult to determine which cells are stained with an anti-VCAM-1 mAb on single color immunohistology slides (Figs. 2C, D). To focus on VCAM-1 expression by HEV endothelia, we stained frozen sections of BALT for PNAd and VCAM-1 by two-color IF (Figs. 2I-N). Quantitative evaluation revealed that 66 ± 11% of BALT HEVs, as identified by high endothelial morphology and expression of PNAd, were PNAd^+ ^VCAM-1^+ ^and 33 ± 11% were PNAd^+^VCAM-1^- ^(*n *= 5 patients).

These results indicate that the HEV adhesion molecules that are available to recruit lymphocytes into BALT differ significantly from those that are available to recruit lymphocytes into PP. Specifically, in BALT, all HEVs expressed PNAd, most HEVs expressed VCAM-1, and no HEVs expressed MAdCAM-1. In PP, all HEVs expressed MAdCAM-1 and a few small HEVs expressed PNAd or VCAM-1.

### Lymphocytes in BALT

**Figure 3:**
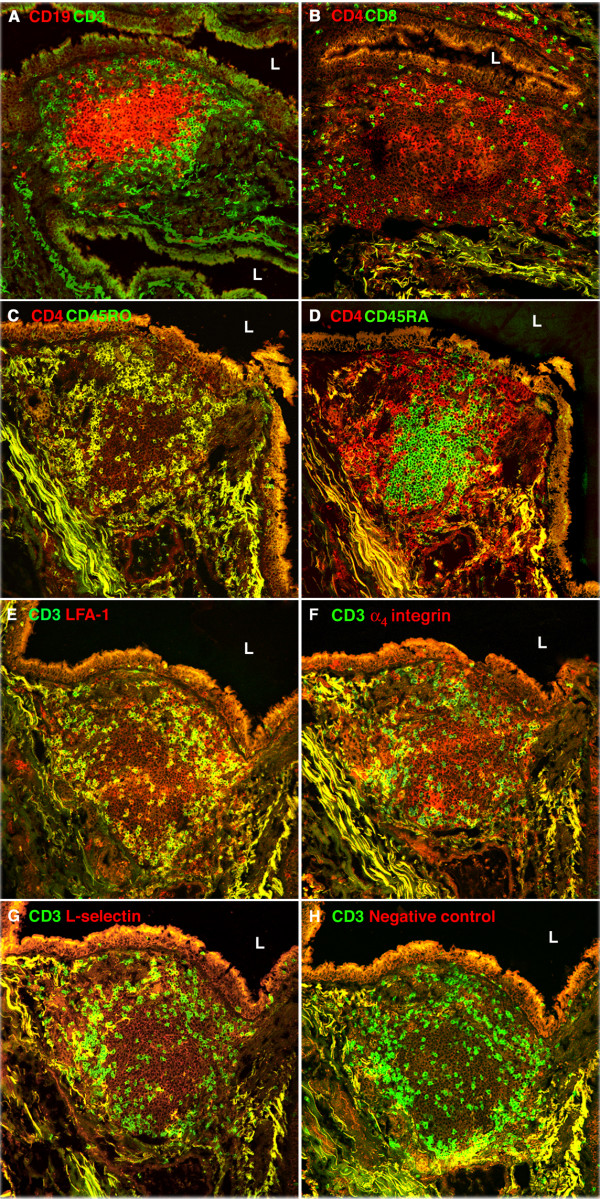
**Lymphocytes in human BALT**. **(A-D)**: Semi-serial frozen sections of BALT were stained with mAbs against CD19 and CD3 (A), CD4 and CD8 (B), CD4 and CD45RO (C), and CD4 and CD45RA (D). L is in the airway lumen of the bronchus. (A) B cell follicle (CD19^+^, red) surrounded by T cells (CD3^+^, green). (B) CD4^+ ^cells (red) outnumber CD8^+ ^cells (green). (C) Almost all BALT CD4^+ ^cells have a memory phenotype (CD4^+^CD45RO^+^; yellow). (D) CD4^+ ^cells with a naive phenotype (CD4^+^CD45RA^+^, yellow) are very rare in BALT. Most cells in the B cell follicle are CD4^-^CD45RA^+ ^(green). **(E-H)**: Semi-serial frozen sections of BALT from a different patient were stained with FITC-anti-CD3 mAb combined with a PE-conjugated mAb against an adhesion molecule or a negative control antigen. L is in the airway lumen of the bronchus. Most BALT T cells, as defined by expression of CD3, also expressed LFA-1 (E, yellow); a minority of BALT T cells expressed α_4 _integrin (F, yellow) or L-selectin (G, yellow). In contrast, most BALT B cells (CD3^-^) express LFA-1 (E, red), α_4 _integrin (F, red) and L-selectin (G, red). The PE-negative control mAb did not stain T or B cells in BALT (H). (two-color immunofluorescence stains. original magnification 200×).

We used two-color frozen section IF staining to determine which lymphocyte subsets are present in BALT, and whether certain subsets occupy specific microenvironments within the tissue. Our stains revealed well demarcated B (CD19^+^) and T (CD3^+^) cell zones (Fig. 3A). The B cells formed central aggregates that were surrounded by T cells, most of which expressed CD4 (Figs. 3A and 3B). CD45RO (memory marker) and CD45RA (naive marker) were expressed on 86 ± 2% and 9 ± 2% of the CD4^+ ^T cells (*n *= 7 patients), respectively (Figs. 3C and 3D).

Next, we examined BALT T cells and B cells for the expression of L-selectin, which binds to PNAd; α_4 _integrin, which is a subunit of α_4_β_1 _integrin (which binds mainly to VCAM-1) and α_4_β_7 _integrin (which binds to MAdCAM-1); and LFA-1, which binds to ICAM-1 and ICAM-2 [1]. Most T cells (96 ± 5, *n *= 6 patients) expressed LFA-1 (Fig. 3E). A minority of T cells expressed α_4_integrin (43 ± 12%) or L-selectin (20 ± 19%) (Figs. 3F and 3G). In contrast, almost all B cells expressed LFA-1, α_4_integrin and L-selectin. BALT lymphocytes did not stain with the negative control mAbs (Fig. 3H). Thus, BALT T cells differ from BALT B cells in expression of adhesion molecules that are involved in lymphocyte migration from the bloodstream into tissues.

## Discussion

Migration of lymphocytes from blood vessels into tissues is a complex process with multiple adhesion and activation steps [1,32]. Briefly, lymphocytes transiently adhere to the endothelial surface of venules in the tissue (step 1), are activated by chemokines (step 2), and adhere firmly to the endothelia (step 3), before migrating through the vessel wall into the tissue [1,32]. Thus, the adhesion molecules that are expressed on the HEVs in a secondary lymphoid tissue are important in determining which subsets of lymphocytes migrate into the tissue. Here we evaluated BALT from lobectomy specimens of 17 human adults with lung carcinoma for the expression of HEV and lymphocyte adhesion molecules that might recruit lymphocytes from blood vessels into BALT. We found that BALT HEVs expressed PNAd, ICAM-1 and ICAM-2, with or without VCAM-1. MAdCAM-1 was not expressed on HEVs in any of the BALT samples. The adhesion molecule profile of HEVs in human BALT is similar to that of HEVs in mouse BALT, differing only in the extent of VCAM-1 expression (66% of HEVs in human and 91% of HEVs in mouse BALT) [9]. Thus, BALT in humans, as in mice [9], has HEV adhesion molecules that are capable of recruiting distinct subsets of lymphocytes from the bloodstream into the tissue.

As in mice, the combination of adhesion molecules on HEVs in human BALT differs from that on HEVs in other secondary lymphoid tissues [9]. Specifically, VCAM-1, which was expressed by endothelia of 66% of BALT HEVs in our study, is not significantly expressed on HEV endothelia in PP (Fig. 2D), adenoids and peripheral LNs [31,33]. PNAd, which was strongly expressed on all HEVs in BALT, is weakly expressed on HEVs in appendix and PP (Fig. 2B) [34]. MAdCAM-1, which was not expressed on BALT HEVs, is strongly expressed on appendix and PP HEVs [30].

We found that 86% of BALT CD4^+ ^T cells had a memory phenotype (CD4^+ ^CD45RO^+^). Since BALT does not have afferent lymphatic vessels [6], these memory T cells may have migrated through blood vessel HEVs into BALT and/or may have arisen *in situ *from the maturation of naive T cells. As in other secondary lymphoid tissues, BALT T cells differ from B cells in adhesion molecule expression. L-selectin, which binds to PNAd, was expressed on 20% of T cells and almost all B cells in BALT. α_4 _integrin, which is a subunit of α_4_β_1 _integrin that binds to VCAM-1, was expressed on 43% of T cells and almost all B cells in BALT. LFA-1, which binds to ICAM-1 and ICAM-2, was expressed on almost all lymphocytes in BALT.

The Stamper-Woodruff *in vitro *binding assay can be used to determine which adhesion molecules mediate the binding of viable lymphocytes to HEVs on frozen sections of tissues [12,35]. Due to the scarcity of frozen samples of human BALT, we were unable to perform these assays. However, our immunohistology studies on human and mouse BALT and our functional studies on mouse BALT [9] suggest that PNAd and VCAM-1 are involved in organ-selective recruitment of specific subsets of lymphocytes to human BALT. Specifically, L-selectin^+ ^naive T cells could be recruited from the bloodstream into human BALT by binding to PNAd^+ ^HEVs [36]. Since specialized cells, such as M cells and/or dendritic cells, transport airway luminal antigens into BALT, the naive T cells could meet antigen-bearing dendritic cells in BALT, resulting in the generation of lung-specific α_4_β_1 _integrin^+ ^memory T cells. Following release into the bloodstream, the α_4_β_1 _integrin^+ ^memory T cells could be recruited back to BALT by binding to VCAM-1^+ ^HEVs. Additionally, the α_4_β_1 _integrin^+ ^memory T cells could migrate into inflamed lungs by binding to VCAM-1, which is highly expressed on vessels in human inflamed lung [37-39]. Thus, in humans as in mice, the VCAM-1/α_4_β_1 _integrin adhesion system may unify the migration pathways of T cells into BALT and inflamed lung.

The frequency of BALT in normal lungs of healthy adults varies considerably between species. For example, BALT is found in lungs of most normal adult rats, rabbits and guinea pigs [5,6,10,12,13,40]. In contrast, the existence of BALT in lungs of healthy adult humans is controversial. In published reports, BALT was found in 0% to 74% of normal human lungs, as compared to 35% of lungs in our study [14-16,18,20,41]. The marked differences in the frequency of human BALT between studies may be due to several factors, including the criteria used to determine if the lung is "normal" and/or the person is "healthy", differences in human subject populations, the source and size of the specimen (i.e., bronchial biopsy, surgical wedge resection, or surgical resection of a lobe or lung), the number of tissue samples and sections examined, and the histologic criteria used to identify BALT.

As with other lymphoid tissues, BALT can undergo marked changes in size, cellular composition, and function during local immune responses. In animal models, BALT can be induced and/or activated by infection or immune stimulation of the lower respiratory tract [23,42-45]. In humans, hyperplastic BALT can be seen in patents with chronic pulmonary inflammatory disorders, such as hypersensitivity pneumonitis, and in patients with autoimmune diseases, including rheumatoid arthritis and Sjogren's syndrome [15,46-50]. Exposure to irritants, such as those in cigarette smoke, may influence the development of BALT, as BALT was found in 82% of smoking and 14% of non-smoking human adults in one study [18]. In our study, BALT was slightly more common in current smokers (42%) than in non-smokers (29%).

It is clear from this study and previous studies that human BALT shares many features with secondary lymphoid tissues, including distinct T cell and B cell zones, follicular dendritic cells, PNAd^+ ^HEVs, and expression of lymphoid chemokines CXCL13 and CCL21 (Fig. 2) [49]. Some unique features of BALT in humans and animals, however, lead to the debate regarding whether BALT is a secondary lymphoid tissue controlled by a precise developmental program or a tertiary lymphoid tissue controlled by lymphoid neogenesis. BALT varies by species, strain, age, and antigen stimulation: it is more common in rabbits and rats than in mice [5,6,10,13,40]; autoimmune-prone nonobese diabetic mice and old mice have more prominent BALT than nonautoimmune-prone and young mice, respectively [9]; and antigen stimulation and microbe infection can induce BALT formation in lungs of mice [23,42,43,51]. Additionally, HEVs in mouse and human BALT express VCAM-1 (Fig. 2) [9], which is frequently seen on HEVs of tertiary lymphoid tissues but not of LNs and PPs [52]. Thus, BALT is not identical to "conventional" secondary lymphoid tissues such as LNs and PPs.

A limitation of our study and of several other studies of human bronchopulmonary immunology [35,53] is that the tissues were obtained from carcinoma lobectomy specimens. The factors, such as cigarette smoke, that led to the development of the carcinoma could also lead to the development of chronic inflammation, such as chronic bronchitis. In addition, the carcinoma could initiate an inflammatory response (for example, by obstructing a bronchus, leading to post-obstructive pneumonia). We made every effort to minimize these potential confounding factors. Specifically, we excluded patients with 1) known inflammatory pulmonary diseases; 2) macroscopic evidence of inflammation or infection in the lobectomy specimen; or 3) histologic features of inflammation or infection [29]. Moreover, we used strict histopathologic criteria to identify "classic" BALT as described by Bienenstock and colleagues and in the pathology literature [5,29].

## Conclusion

In summary, we found that the HEVs in human BALT express a unique set of adhesion molecules that are very similar to those expressed in mouse BALT but markedly different from those expressed in other human secondary lymphoid tissues. The adhesion molecules on human BALT HEVs may be important in recruiting specific subsets of naive and memory/effector lymphocytes to BALT, where they may be involved in the development of bronchopulmonary immune responses to inhaled antigens and pathogens. Thus, BALT may be involved in the induction or/and modification of physiologic and pathologic bronchopulmonary immune responses. Selective targeting of PNAd/L-selectin- and VCAM-1/α_4_β_1 _integrin-mediated lymphocyte migration pathways might serve as a novel strategy for treating inflammatory bronchopulmonary diseases.

## List of abbreviations

BALT: bronchus-associated lymphoid tissue; HEVs: high endothelial venules; Hr: hour; IF: immunofluorescence; LNs: lymph nodes; mAb: monoclonal antibody; MAdCAM-1: mucosal addressin cell adhesion molecule-1; Min: minutes; PBS: phosphate-buffered saline; PNAd: peripheral node addressin; PPs: Peyer's patches; SD: Standard derivation; VCAM-1: vascular cell adhesion molecule-1

## Competing interests

The authors declare that they have no competing interests.

## Authors' contributions

NK and BHX contributed equally as co-first authors. SAM and TM contributed equally as co-senior authors. NK, BHX, SAM and TM designed the project, analyzed and interpreted the data, and wrote the manuscript. HN performed the lobectomies, collected and processed specimens, obtained written informed consent from all patients, and participated in data interpretation. NK, BHX, KA and MK performed experiments. CT and TT participated in data analysis and interpretation. All authors read and approved the final manuscript.
